# Applications of Nanotechnology in Sensor-Based Detection of Foodborne Pathogens

**DOI:** 10.3390/s20071966

**Published:** 2020-04-01

**Authors:** Harsh Kumar, Kamil Kuča, Shashi Kant Bhatia, Kritika Saini, Ankur Kaushal, Rachna Verma, Tek Chand Bhalla, Dinesh Kumar

**Affiliations:** 1 School of Bioengineering & Food Technology, Shoolini University of Biotechnology and Management Sciences, Solan 173229, H.P., India; microharshs@gmail.com (H.K.); sainikritika@gmail.com (K.S.); 2 Center for Basic and Applied Research, Faculty of Informatics and Management, University of Hradec Kralove, Hradec Kralove 50003, Czech Republic; 3 Department of Biological Engineering, College of Engineering, Konkuk University, Seoul 143-701, Korea; shashikonkukuni@konkuk.ac.kr; 4 Centre of Nanotechnology, Amity University, Manesar, Gurugram, Haryana 122413, India; ankur.biotech85@gmail.com; 5 School of Biological and Environmental Sciences, Shoolini University of Biotechnology and Management Sciences, Solan 173229, H.P., India; rachnaverma@shooliniuniversity.com; 6 Department of Biotechnology, Himachal Pradesh University, Summer Hill, Shimla 171005, H.P., India; bhallatc@rediffmail.com

**Keywords:** nanotechnology, nanomaterials, safety, sensor, foodborne pathogens

## Abstract

The intake of microbial-contaminated food poses severe health issues due to the outbreaks of stern food-borne diseases. Therefore, there is a need for precise detection and identification of pathogenic microbes and toxins in food to prevent these concerns. Thus, understanding the concept of biosensing has enabled researchers to develop nanobiosensors with different nanomaterials and composites to improve the sensitivity as well as the specificity of pathogen detection. The application of nanomaterials has enabled researchers to use advanced technologies in biosensors for the transfer of signals to enhance their efficiency and sensitivity. Nanomaterials like carbon nanotubes, magnetic and gold, dendrimers, graphene nanomaterials and quantum dots are predominantly used for developing biosensors with improved specificity and sensitivity of detection due to their exclusive chemical, magnetic, mechanical, optical and physical properties. All nanoparticles and new composites used in biosensors need to be classified and categorized for their enhanced performance, quick detection, and unobtrusive and effective use in foodborne analysis. Hence, this review intends to summarize the different sensing methods used in foodborne pathogen detection, their design, working principle and advances in sensing systems.

## 1. Introduction

Nanotechnology is the science to understand the matter at a nanoscale dimension, generally ranging between 1 and 100 nm. This technology involves the fabrication, manipulation, study of technique, material, modes and use of nano-devices in various applications. Nowadays, nanotechnology is extensively used in developing biosensors using different types of nanocomposite [1,2,3]. The different nanomaterials used in nanotechnology display unique features including chemical, physical and surface effects because of their dimensions and tolerances to sizes less than 100 nanometers. 

Every year globally, contaminated food is responsible for 420 000 deaths and 600 million cases of food-borne infection. According to WHO, approximately 30% of death occurs among children (≤5 years) due to foodborne disease [4]. The established microbial culture methods may help in the detection of a single specific bacterium, but this procedure takes a few days for results, and in many instances these methods do not produce considerable data. By using nanotechnology-based methods, the pathogenic bacteria present in complex food products can be detected with high sensitivity and specificity as compared to the conventional methods [5,6,7]. A biosensor is a device in which a biological element (i.e., an antibody, receptor, nucleic acid, or other biorecognition element) interacts with an analyte and the response thus generated is transformed into an electrical signal by a transducer. The response of biosensors is highly specific, quick, free from interference, and has low limits for detection, but still the non-targeted microorganisms can interfere with the operations of biosensors. 

The development of many signal transduction technologies using nanomaterials in biosensor is transforming the field of biological and chemical analysis to empower in vivo studies. In microbial detection, highly fascinating and promising outcomes have been produced in research using nanotechnology. The characteristics of nanomaterials with high surface permeability, higher surface to volume ratio, reactivity, and high penetrability allow less material and substrate usage and as compared to large size materials, they work more efficiently in physical and chemical reactions [8]. A high advantage of establishing a small automated system was worked out for the use in field with rapid and cost-effective microbial detection, and sensitive stick tests were developed by using gold nanoparticles with high specificities for the same [9,10,11]. This review highlights the effectiveness of nanomaterials for developing biosensors, especially for detecting the microbial pathogens present in contaminated food. The first section of this review describes the various properties of the nanomaterials used for sensing applications, and the safety issues and guidelines issued by various organizations for use at workplace or in laboratories. The other section discusses the utilization of nanomaterials and present status of sensors used for the detection of foodborne pathogens in various food matrixes. 

## 2. Criteria for Selection of Nanomaterials for use in Biosensors 

Nanosensors are generally used for the measurement of biological response output quantity as well as for conversion of biological response into output signals for further interpretation and analysis. The selection of nanomaterial used in various biosensing applications is dependent on its properties (Table 1). 

**Figure 1:**
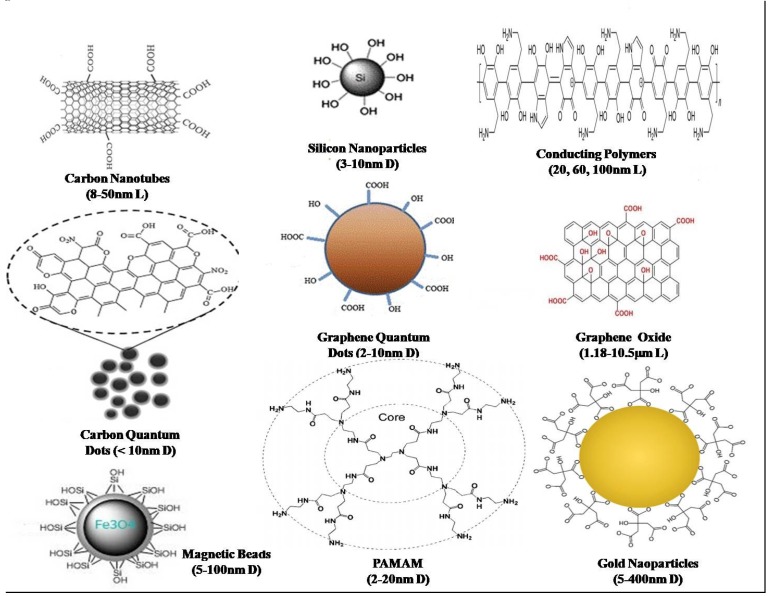
Commonly used nanomaterials in various kind of sensors fabrication with their sizes. L: length; D: Diameter.

Nanomaterial-based sensors are highly sensitive and specific in their nature as compared to the traditional material-based sensors. Some common nanomaterials used in sensor technology are shown in Figure 1.

The nanosensors are operated at a scale similar to the biological processes to increase the specificity of biological response. One of the important steps of experimental design before addition of a nanomaterial into the sensing application is “Nanofabrication”. This step leads to two important actions, i.e., first, the manufacturing and designing of nanoscale adhesive through the use of integrated circuits and second, the use of micromachining processes for the engineering of nanomaterial surfaces. Four basic processes involved in biosensing by nanofabrication are chemical bonding, photolithography, surface etching strategies and thin film etching/growth. With the rise of chronic diseases, e.g., diabetes and cancer, there is a need for awareness among the masses for the early-stage identification of these diseases, as biosensors provide a quick response and high sensitivity at the initial stages. Nanoscale electrodes are produced from the lithography technique, which is one of the basic process of nanofabrication. The use of nanomaterials in biosensing provides better and greater surface area for immobilization with better precision [21]. Glucose biosensors were first developed using this technique by the use of the enzyme glucose oxidase and the detection of analytic materials was improved by the addition of platinum nanoparticles above the sheets of carbon nanotubes. These biosensors enable the recognition of glucose from several sources other than blood. The antigen–antibody complexes formed in the reaction can also be identified by immune-sensors in which thin films are coated above the sensing surface to allow for quick and better recognition of the analytes [22].

Nanoelectromechanical systems (NEMS) are other highly sensitive devices with nanoscale functions. This technology, when engineered with nanomaterials, provides novel properties from nanoscale to nanometer level in energy harvesting, sensing display, imaging, portable power generation and also in drug delivery. NEMS, along with micro-electrochemical systems (MEMS) devices, result in better performance with mechanical materials when attached to biological systems and lead to improvement of bio-adhesion features and response to a widespread range of stimuli. This technology enables the demonstration of the biochemical interactions with the help of biomarkers for the rapid diagnosis of new diseases [23]. Flurophores are other important components of biosensors which can absorb and emit light within wavelengths of excitation and emission spectra and make use of total internal reflection. These are commonly used as recognition reagents in flow cytometry in conjugation with the antibody critical angle of reflection.

In nanomaterials, the sensing materials should have sharp and precise scale optical response with the incident light surface plasmon resonance (SPR) [24]. The SPR furnishes the nano-biosensor in a better way and allows the estimation of biological interactions through the detection of the minutest interaction phenomenon. The plasmonic peak of nanoparticles with sizes smaller than 10 nm can be described in a better way by the modified dielectric function as compared to the other models [25,26,27]. The safety of the environment and human health is of prime concern and many reports have highlighted the toxicity associated with various nanomaterials (Table 2).

Many organizations across world have given recommendations regarding the safety of researchers and employees dealing with nanomaterials at the workplace (Table 3).

## 3. Various Nanomaterials used for Sensor Fabrication with Special Reference to Food Borne Pathogens 

### 3.1. Carbon Nanotubes 

In the last decade, carbon nanotubes (CNTs) have emerged as one of the most extensively used nanomaterials in drug delivery and in biomolecular techniques. CNTs are cylindrical hollow tubes containing one or more concentric layers of graphite enclosed by fullerenic hemispheres, referred to as single- to multi-walled CNTs, respectively. They have unique structures, high electrical and mechanical properties, chemical stability, light weight, high thermal conductivity, unique electrocatalytic action, minimal surface fouling and high surface to volume ratio [47].

The key challenge for developing effective biosensors is their specificity, sensitivity and detection time to assess the presence of food-borne pathogens in normal and toxin-supplemented samples. Single-wall surface carbon nanotubes (SWCNTs) were employed in a DNA sensor application for the detection of *Salmonella* using N-ethyl-N’-(3-dimethylaminopropyl) carbodiimide hydrochloride (3-dimethylaminopropyl) covalently bonded to the nanotubes [48]. For this, an electrode along with ssDNA probe solution (*Salmonella* specific) was incubated at room temperature for 2 h. The sensor showed sensitivity at the target concentration of 1 × 10^−9^ mol/L DNA. Moreover, no fluctuation was noted in the signal via the ssDNA probe on the mismatching of the single nucleotide. An amino-modified aptasensor was developed using indium tin oxide (ITO) deposited on a multi-walled carbon nanotube (MWCNTs) electrode and assessed for the detection of *Salmonella enteritidis* and *Salmonella typhimurium* with detection limits of 5.5 ×10^1^ and 6.7 × 10^1^ cfu mL^−1^, respectively [49]. Additionally, the sensitivity of an aptasensor was validated via the PCR technique, by detecting the 148bp invA gene, which is also present in *S. enteritidis* and *S. typhimurium*. The sensitivity limit of PCR was found to be 10^2^ cfu mL^−1^ for both the strains of *Salmonella*.Meanwhile, the sensitivity limit of the aptasensor was found to be 10^1^ cfu mL^−1^ when assessed on raw chicken samples which were spiked with *Salmonella*. Hence, this confirms that the ssDNA/MWCNTs/ITO electrode coupled to an aptamer is more effective in comparison to PCR detection approach on the basis of its detection limit. Furthermore, the developed sensor shows no specificity against non-*Salmonella* bacteria. In 2014, the group of researchers immobilized anti-*Escherichia coli* antibodies on SWCNT-based joint biosensor developed to detect the *E. coli* K-12 strain. The developed sensor showed the detection limit of 10^2^ cfu mL^−1^ in less than 5 min. Moreover, minute fluctuation in the electric current was detected, when checked with *Staphylococcus aureus*, which confirms its specificity and sensitivity to *E. coli* [50].

### 3.2. Gold Nanoparticles 

Gold nanoparticles are frequently used in nanotechnology-based applications. Gold nanoparticle (AuNPs) synthesis in organic or aqueous solvents requires a stabilizing mediator (surfactant) for its stability and can be achieved by chemical binding or by adsorption of the appropriate mediator on the gold NPs. Usually, to avoid the aggregation of loaded NPs, the surfactant need to be loaded and different gold NP properties can be adjusted by choosing separate surfactants [51].

The biological compatibility, exceptional conducting capability, and high surface-to-volume ratio are the few characteristics of AuNPs, which makes it the nanoparticle of interest [52]. The redox activity is an interesting characteristic of gold NPs, which enhances the sensitivity of electrochemical biosensors in the analysis of foodborne pathogens. The use of gold NPs onto electrochemical biosensors in conjugation with ssDNA complementary to the microbial DNA under evaluation improves their binding with DNA-gold NPs on the transducer surface and enhances the sensitivity of the developed biosensor [53]. In another example, gold NPs were used in conjugation with redox enzymes that precisely oxidize/reduce the analyte as the substrate in the reaction. On binding to the analyte, the immobilized enzyme-gold NPs eventually increase the current signal and confirm detection [51]. 

The AuNPs conjugated with *E. coli* O157: H7 antibodies were also used for detection of *E. coli* O157: H7 in milk. In this study, screen-printed carbon electrodes (SPCE) were fabricated with AuNPscontaining *E. coli* O157: H7-specific antibodies conjugated with horseradish peroxidase with a detection limit of 10^2^–10^7^ cfu mL^−1^ using hydrogen peroxide and ferrocene dicarboxylic acid (FeDC) as a substrate [54]. This analysis confirmed that immunosensing strips of AuNPs/FeDC–SPCE shows no specificity towards other bacteria like *E. coli* K12, *Listeria monocytogens*, *S. choleraesuis* and *Vibrio parahaemolyticus*. Hong et al. developed a selective and quick electrochemical biosensor for the detection of *Norovirus* (NoV) [55]. This biosensor is fabricated with a nanostructured gold electrode containing concanavalin A (ConA) as a recognition element that selectively detects NoV. In this study, the sample solution extracted from lettuce was used to measure the concentration of NoV in a realistic atmosphere for its validation with a detection limit of LoD=60 copies mL^−1^. The sensor was found to be non-targeted against hepatitis A viruses (HAV) as well as hepatitis E viruses (HEV). It also showed effective thermal stability at both 4 and 25 °C, respectively.

Davis et al. developed a modified electrode-AuNPs biosensor for assessing the presence of *L. monocytogenes* in spiked blueberries and the detection limit was found to be 2 log cfu/g after 1 h of assay [56], whereas, another group of researchers developed an enhanced AuNP aptasensor via Surface-enhanced Raman spectroscopy (SERS) for instantaneous detection of *S. aureus* and *S. typhimurium* in a spiked sample of pork [57]. In this study, the intensities of the signals for other bacteria like *Bacillus cereus, E. coli, Shigella dysenteriae* and *V. parahaemolyticus* were found to be very low. The outcome of this aptasensor was similar to the plate counting method exhibiting recovery between 108.33% for *S. aureus* and 94.12% for *S. typhimurium*.

### 3.3. Quantum Dots 

Carbon quantum dots (CDs) are very small artificial semiconductor particles with sizes normally less than 10 nanometers. These nanoparticles are extensively used in research due to their high luminescence properties, high solubility and biocompatibility [58,59]. Carbon quantum dots (CDs) are quasi-sphere nanoparticles (diameter less than 10 nm) formed from crystalline sp2 hybridization graphite cores and amorphous aggregations used in bioanalytics and biolabeling. On the other hand, graphene quantum dots (GDs) consist of single or very few graphene lattices (<10). Due to bigger conjugated domains and periodic structure, the GDs are generally more crystalline than CDs. These nanostructures based on carbon are actually two distinct allotropes and both the allotropes are functionalized with oxygen-related complex surface group molecules such as carboxylates or hydroxylate derivatives that improve optical features and particle solubility [60,61]. The variability in fabrication of these materials gives rise to diverse surface functionalization and more complex hybridization in biosensor applications.

CDs have been used to detect harmful microbial culture and toxins, together with *S. typhimurium* and aflatoxin B1 (AFB1) [62,63]. Wang et al. fabricated carbon dot aptamer complexes (CD-apt) for the quantitative identification of *S. typhimurium* in eggshell and tap water solutions at a test range of 10^3^ to 10^5^ cfu mL^−1^ and a LOD of 50 cfu mL^−1^, for which the detection time was found to be 2 h without interference from *E. coli* O157:H7 and *S. aureus* [62]. The developed sensor exhibited unvaried results in comparison to the standard plate count method in the egg sample as well as in tap water the standard plate count showed 3.6 × 10^4^ and 5.9 × 10^4^ results whereas for the developed sensor the readings were 3.27 × 10^4^ and 5.51 × 10^4^ cfu mL^−1^, respectively. Various trials have verified the specificity of microbial identification using biosensors in simple or complex food settings using CDs, GQDs, carbon nanotubes and semiconductors [63,64,65]. Wang et al. assembled CDs and AuNPs for specific AFB1 detection with the help of an aptamer and achieved a 5 pg/mL LOD (16 pM) [63]. This method was employed with actual samples like corn and peanuts, which were supplemented with varied concentrations of AFB1 in which average recovery was found to be in range of 92%–105%. This recovery enhanced on changing the aptamer, such as ochratoxin aptamers (designed to assess fungal toxins only). 

### 3.4. Magnetic NPs beads as Label in Biosensor-based Detection

Magnetic NPs are another class of nanomaterials used in biosensors which can be amended by changing the magnetic field. These NPs are clusters of magnetic beads of 50–500 nm diameters [66,67,68]. Magnetic NPs have emerged as one of important fabricating material to develop a flow assay as they have strong color and can separate the target material from the complex matrix. These NPs have additional benefits as it provides robust magnetic and visual signals. Various researchers have used magnetic beads to develop flow assays for detecting pathogenic bacteria [69,70]. Wang et al. developed antibody-covered magnetic beads of 300nm and used them as indicators for spore detection of *Bacillus anthracis* with a detection limit of 6 × 10^4^ spores/g of milk powder, 2 × 10^5^ spores/g of starch, and 5× 10^5^ spores/g of baking soda, respectively [71]. The designed sensor did not show any specificity towards other *Bacillus* species like *B. cereus, B. thuringiensis* and *B. mycoides*. In contrast to traditional lateral-flow method, this process does not require the pre-treatment of the sample and it provides instant results for magnetic, naked-eye and optical detection within 20 min. Suaifan et al. established a biological assay with magnetic beads for robust recognition of *E. coli* O157:H7 in food with detection limits of 12 cfu mL^−1^ of broth and 30–300 cfu mL^−1^ of other food matrices [72]. In this study, peptides of *E. coli* O157:H7 (e.g., protease) were used asa substrate and conjugated with magnetic nanoparticles (MNPs). Furthermore, the crude protease synthesized by *E. coli* O157:H7 was down-streamed onto immobilized sensing platform and during enzymatic reaction a magnet attached on the back of sensor stripe, magnetizingthe cleaved MNP-peptide moieties, and generating the visual signals for qualitative assessment of the test sample within 30 seconds. The developed biosensor exhibited long-term stability, i.e., six months, and showed no specificity towards *L. monocytogenes, Pseudomonas aeruginosa* and *S. aureus* protease. On the other hand, Xia et al. developed gold magnetic nanobeads for the rapid recognition of *S. choleraesuis* with a detection limit of 5 × 10^5^ cfu mL^−1^ and detection time of 20 h in whole milk as compared to colloid gold-based lateral flow assay with a limit of 5 × 10^6^ cfu mL^−1^, thus confirming the superiority of magnetic beads to the colloidal gold [73].

### 3.5. Dendrimers 

Dendrimers (DEN) are complex globular shaped-branched structures of 2–20 nm in size. The structural properties like monodispersity, manageable size, easily amendable surface functionalities, hydrophilicity, high mechanical and chemical strength makes them the preferred synthetic nanoparticle for developing biosensors [74]. The polyamidoamine (PAMAM) dendrimer is one of these which hasgained significant attention as it provides large surface areas with high number of functional groups to allow the easy binding of biological entities. It also contains mono-disperse as well as hyper-branched polymers with active functional groups present at end of dendrimer structure. These functional groups aid in immobilizing the bio-recognizing molecules, by acting as a bio-conjugating moiety and play diverse roles in biosensor technology. Electrochemical techniques like amperometric, electrochemiluminescence, impedimetric and potentiometric are generally used for estimating specific molecules using dendrimers with high selectivity and sensitivity [74]. 

Shiddiky et al. developed the competitive and sandwich-based bioassays for assessing DNA and protein using H_2_O_2_ reduction activity using a conducting polymer based on poly-5,2′:5′,2″-terthiophene-3′-carboxylic acid (pTTCA) [75,76]. Another biosensor, made up of the monolayer of 3G PAMAM (poly amido amine) dendrimer covalently linked by chemisorption to AuNPs/CdS nanoparticles was also developed using immobilized AuNPs on bioreceptor molecule and showed a detection limit of 450 aM and 4 fg mL^−1^, respectively, for DNA and protein. This biosensor was 70 times more sensitive as compared to plain pTTCA layer due to AuNPsbeing attached on the pTTCA/DEN layer, which allows the binding of different proteins, avidin and hydrazine. Competitive immune interaction-based sensors have also been developed for detecting anti-microbial agents and biomarkers. The detection of AFB1, a food contaminant, was done using an aptamer-based biosensor [77]. In this study, the fourth generation polyamidoamine dendrimers were immobilized on a cystamine-covered gold electrode with further attachment with AFB1-specific DNA aptamers and showed a detection limit of LOD = 0.40 ± 0.03 nM at 4 °C without losing its stability for up to 60 hours. The developed sensor did not exhibit any specificity towards ochratoxin A (OTA) and was effective in detecting the AFB1 in contaminated peanuts sample.

### 3.6. Silicon Nanomaterials

Silicon nanostructure-based sensors have been developed with high specificity for use in rational fabrication biosensing and bioimaging applications [78,79,80]. Silicon nanomaterials can be converted into molecules knows for renal clearance as these are biodegradable in nature and are excreted from the body without any toxicity evidence [81,82]. Silicon nanoparticles (SiNPs), with a diameter of 3–10 nm, have been permitted by the Food and Drug Administration for use in human clinical trials [83]. A variety of silicon surface-enhanced Raman scattering (SERS) sensors are used in selective and sensitive detection of reproducible chemical and biological species. Pathogens with colony forming units of *E. coli* can be detected using biosensors based on porous silicon using chemiluminescence assay [84]. The sensitivity of this biosensor chip for *E. coli* was determined to be 10^1^ and 10^2^ cells for 40 and 30 min, respectively. In another study, porous silicon was fabricated using an anodization process in an electrochemical Teflon cell [85]. In this study, platinum wire was used as a cathode and the silicon chip was used as the anode and ssDNA of *S. enteritidis* strain was used as a probe, for functionalized porous silicon platform. It was further observed that biosensors with porous silicon were highly sensitive and have more active surface area as compared to the biosensors based on planner silicon and probes specific to the targeted DNA.

### 3.7. Graphene-based Nanomaterials

Graphene-based nanomaterials are another type of material used as transducers of biosensors. These nanomaterials are generally used for the conversion of targeted and receptor molecules for detectable measurement using EDC/NHS chemistry [86]. On the other hand, graphene is the most commonly used nanomaterial for different biosensors designs with different transduction modes as it contains a large surface area, capacity to immobilize with different molecules with high electron transmission rate and electrical conductivity [87]. Graphene-based nanomaterials can also be used as a quencher to generate fluorescent transducer-based biosensors, as reduced graphene oxide (rGO), graphene (G), and graphene oxide (GO) possess a very high fluorescent quenching efficiency [88,89,90]. During sensor design, the detection limit of targeted molecules is affected by graphene and the sensitivity and selectivity of biosensors can also be affected by bioreceptors and G, GO or rGO sheet orientation. Differences can be observed in the sensing performance of biosensors by functional groups, graphene oxidation state, and number of layers as well as by different derivatives used. 

A GO-modified iron oxid-chitosan hybrid nanocomposite-based electrochemical sensor was also used for *E. coli* O157:H7 detection using a specific probe oligonucleotide sequence covalently immobilized on nanocomposite films [91]. This sensor showed a detection limit of 1 × 10^−14^ M with a linear response to the complementary DNA in 10^−6^ to 10^−14^ M. Meanwhile, the specificity of the pDNA/GIOCh/ITO bioelectrode against various target DNA sequences (complementary, non-complementary and one base mismatch) and with the samples of *E. coli, S. typhimurium, Neisseria meningitidis* and *Klebsiella pneumonia* showed insignificant signal. This confirms that the fabricated biosensor has high selectivity, sensitivity and reserved significant activity (i.e., 90% of the initial activity) even after the usage of up to sixcycles. In another study involving a graphene-based biosensor designed specifically for detection by *E. coli* O157:H7-specific antibodies showed sensitivity in range of 10–100 cells ml^−1^ and did not display any specificity towards *E. coli* DH5α strain [92]. The shelf-life of functionalized chips of this biosensor when stored at 4 °C lasted for 4 weeks. Srivastava et al. developed an electrochemical sensor for the food toxin detection by binding the monoclonal antibodies on rGO surface to AFB1 with a sensitivity of 68 uA ng^−1^ ml cm^−2^, and a limit of detection of 0.12 ng mL^−1^ [93]. The storage stability of this immune-electrode was determined by passing the current at regular interval of 5 days for 45 days along with 25ng dL^−1^ AFB1 and showed no substantial variation in the current, even after 45 days. In another study, a BSA/anti-AFB1/AuNPs/rGO nanocomposite-based immunosensor showed a high sensitivity of 182.4 µA ng^−1^ mL^−1^ cm^−2^ for the recognition of AFB1 with a limit of detection of 0.1–12 ng^−1^ mL^−1^ and consistency up to 56 days [94].

### 3.8. Conducting Polymers 

Conducting polymers withdistinctive characteristics have made these an effective alternative for some materials currently used in the biosensor fabrication. Polymers are good insulators and some polymers are found to have good conducting properties due to their combination of metallic and semiconductor characteristics. There are varieties of conducting polymers used in different applications [95]. Out of these, polyaniline, polythiophene, and polypyrrole are used as nanomaterials and show biocompatibility and can reduce the leading disturbances affecting the working environment and these also help in preventing the electrodes from fouling [96,97]. Only polyaniline and polypyrrole are extensively used for the detection of foodborne pathogens. Conducting polymers are used as an excellent immobilizing platform with biomolecules at electrodes to deliver better signal transduction, high sensitivity, selectivity, durability, biocompatibility and flexibility [98,99]. 

Tully et al. reported direct immunosensor use for the identification of a cell-surface protein on *L. monocytogenes* with label-free immunosensing of Internalin B (InlB) with a limit of detection for InlB at 4.1 pg mL^−1^ [99]. Muhammad-Tahir and Alocilja reported the performance of a biosensor based on electrochemical transducer using polyaniline in measuring an immune reaction for detecting 7.8 × 10^1^ cfu/mL of *E. coli* O157:H7 in 10 min [100]. Polyaniline was also reported as an identifier for the electrochemical sandwich immunoassay of *E. coli* O157:H7 detection in fresh produce such as lettuce, alfalfa sprouts, and straw-berries and with an average of 81 cfu mL^−1^ in nine samples in 6 mins [101]. Sheikhzadeh et al. used a label-free impedimetric biosensor for the detection of *S. typhimurium* based on the effect of the aptamer/target response to the central conjugation of poly [pyrrole-co-3-carboxyl-pyrrole] copolymer-based aptamer to its electrical characteristics [102]. In this study, the *S. typhimurium* was detected with high selectivity over other pathogens at a concentration range of 10^2^–10^8^ cfu mL^−1^ with a limit of detection of 3 cfu mL^−1^. The developed aptasensor showed high selectivity towards *S. typhimurium* on comparing its detection value with other potential model strains viz. *E. coli* 375, *E. coli* 797, *E. coli* 3274, *Enterobacter*, and *Citrobacter*. Moreover, the developed aptasensor showed robust detection of *S. typhimurium* in 45 min in spiked apple juice.

## 4. Current Status and Future Prospects 

Various developments in nanotechnology have shown its proficiency in detecting pathogenic microbes and resulted in an answer to different problems related to biotransformation and metabolism of ingested NPs (Table 4).

## 5. Conclusions

The application of sensors in food processing industries has also changed the current trend as these can identify the various contaminants formed within the food chain with high sensitivity. The advancements in diagnostics have increased the demand for portable devices for robust and precise detection in food industries. Nano-sensors have the potential to meet both the demand of miniaturization and low-cost analytical devices. In the past few years, applications of e-nose technologies have come through advances in sensor design, material improvements, software innovations and progress in micro-circuitry design and systems integration. There is significant interest in methods for the early detection of quality changes in food products. The development of electronic nose technology has stimulated interest in the use of characteristic volatiles and odors as a rapid, early indication of deterioration in food quality [121,122]. The research on nanotechnology has progressed so much that it has encouraged the expansion of nanosensors for the detection of foodborne pathogens with high improvements overthe conventional methods. Ultrasensitive transglutaminase-based nanosensors used for early diagnosis of celiac diseases in human and identification of foodborne pathogens and food-related disorders using biosensor are some specific examples of biosensor use [123,124,125]. The extensive research progress in nanotechnology for nanomaterial exploration and the development of new mechanisms in the future will enable researchers to develop highly sensitive, specific and unobtrusive nanosensors for analyzing food-borne microbes at an affordable cost. 

## Figures and Tables

**Table 1:** Properties, and synthesis of different types of nanomaterials used in sensors fabrication and other applications.

Nanomaterials	Physical Properties	Synthesis	Applications	Ref
Gold	Redox activity Surface-enhanced Raman scattering (SERS) Surface Plasmon resonance (SPR) Fluorescence quenching	Solution-based approaches	Sensing: electronic devices and electrochemical sensing Therapeutics: drugs delivery Imaging: cell imaging, target tumor specific antigens	[12]
Carbon Nanostructures	Equilibrium structure Lattice constant viz lattice parameter, density, interlayer spacing Optical properties viz fundamental gap Electrical transport Thermal transport Elastic behaviour	Chemical vapour deposition technique Laser- ablation technique Carbon arc-discharge technique	Biomedical applications: artificial implants, tissue engineering, cancer cell identification and drugs and genes delivery Electrochemical devices: supercapacitors and electromechanical actuators used in robots and hybrid electric vehicles Hydrogen storage: fuel cells that powers electric vehicles and laptop computers Field emission devices: lamps, gas discharge tubes, microwave generators Sensors and Probes	[13,14]
Magnetic Nanoparticles	Magnetic effect due to spinning electric- charged particle Critical size depends on magnetic saturation, strength of crystal, exchange forces, surface energy, and shape of the particles Zero coercivity	Co-precipitation Microemulsion Thermal decomposition Solvothermal Sonochemic Microwave assisted Chemical vapour deposition Combustion synthesis Carbon arc Laser pyrolysis	Industrial applications: used as synthetic pigments in ceramics, paints and porcelain Biomedical applications: used *in vivo* to destroy the pathological cells by hyperthermia, drugs delivery, NMR imaging, bioseparation of specific biological entities from their native environment Environmental applications: removal of organic and inorganic pollutants	[15]
Silicon Nanomaterials	Optical properties viz bright emission, photostability, size dependent and wavelength tuneable luminescence and long fluorescence Electronic properties viz quantum confinement, type of dopant, composition of material, surface functionalization and post treatment	Pulsed laser ablation Heating degradation Ball milling Chemical synthesis Electrochemical etching	Light- emitting applications: multicolour silicon-based light emission diodes Energy and Electronic fields: lithium battery, solar cell battery, Microwave assisted filed-effect transistor Photocatalysts	[16]
Graphene oxide	Mechanical properties to enhance the strength Electrical properties include high electron mobility and electrical conductivity Thermal properties	Bottom-up approach Top-down approach	Membranes and Coatings: gas transport, water treatment Stimuli-responsive materials: humidity actuation, thermal/light responsive actuation, electrochemical actuation, multi-stimuli actuation Corrosion resistance Energy storage: lithium ions batteries, supercapacitors	[17]
Dendrimers	Low viscosity High solubility and miscibility due to many chain ends High surface areas in relation to volume Encapsulate guest molecule in the macromolecular interior due to their globular shape	Divergent method Convergent method	Biomedical field: drugs and genes delivery, photodynamic therapy, enhancing drug solubility Water purification Analytical devices	[18,19]
Conducting polymers	High conductivity viz. reversible redox Nonlinear optical properties Electric properties Microwave absorbing properties Wettability	Chemical method Electrochemical method Photochemical method Concentrated emulsion method Inclusion method Plasma polymerization Pyrolysis method	Electronic devices: light emitting diodes, solar cells Electromagnetic shielding materials Microwave absorbing materials Rechargeable batteries Sensors	[20]

**Table 2:** Toxicity studies of various used in sensor development under *in vitro* and *in vivo* conditions.

Nanomaterials	Toxic Effects	Dosage Level	Ref
Multiwalled Carbon nanotubes	Damage to micronucleus, macronucleus, and membrane was observed in *Stylonychiamytilus* Decrease in maternal, fetal weight and skeletal malformation in mouse model Increased abortion rate in mouse model Increase in C-C motif ligand 20, basic fibroblast growth factor, and soluble IL-1 receptor II in human subjects	1 mg/ml 100.8–162.5 µg/mouse 4–20 mg/kg 45 µg/m^3^	[28,29,30,31]
Singlewalled Carbon nanotubes	Fetal morphological abnormalities in mouse model Increased resorption rate in mouse model	0.1–30 µg/mouse 10 mg/kg	[32,33]
Quantum dots (Cadmium telluride)	Reduction in phagocytic activity and hemocyte viability in the hemolymph of *Elliptio complanata* Reduced survival rate in mouse model	8 mg/L 20–125 µg/mouse	[34,35]
Gold NPs	Induced decreases in body weight, red blood cells, and hematocrit in mouse model	550–2200 µg/kg	[36]
Polypyrrole	Cytotoxic for human jurkat cell line, mouse embryonic fibroblasts and mouse hepatoma cell line (MH-22A)	>19.4 µg/mL	[37]
Graphene oxide	Toxic for the liver, kidney, spleen, lung, intestine, and brain in rat model	500 mg/kg	[38]
Magnetite	Acute inflammation in the liver and tarsal joints, induced the vaginal secretion IgA, Bcl-2 reactivity in the hepatocytes in mice model	45 mg/mouse	[39]
Dendrimers (PAMAM)	Increases in lysosomal activity of HaCaT cells, an immortal non-cancerous human keratinocyte cell line	1.5–1.8 µM	[40]

**Table 3:** Guidelines/recommendations to address the safety of nanomaterials used in sensor fabrication.

Country/Agency	Key Guidelines/Recommendations	Applicable Sector	Ref
Australia/University of Wollongong	Eliminating worker exposure to nanomaterials wherever possible throughout the manufacturing and handling of nanomaterials Substitution is unlikely to be an applicable hazard reduction method because the unique properties of nanomaterials are the key to their potential and are essentially driving their development In addition to taking into account the regulatory requirements and production imperatives, safe layouts must be designed to eliminate situations involving risks for the process and for the workers	Small scale laboratory	[41]
India/DST	Nanoparticles are to be handled in a form that is not easily airborne, such as in solution or on a substrate. Use of respiratory air filters N100 or N95 is recommended Wear safety glasses, goggles, full facepiece respirator (Recommended when there is exposure to solvent or hot material)	Research laboratories Industries	[42]
Canada/Concordia University	Awareness or safety training for students, staff, employees or anyone involved working with nanoparticles Development and application of standard operating procedures (SOPs) when working with specific nanoparticles	Laboratory facilities	[43]
European Commission	Operations which involve the likely release of manufactured nanomaterials (MNMs) into the air should be performed in contained installations or in facilities that can be operated remotely from a protected area Processes where there is a potential for creating dusts or aerosols of MNMs should be carried out in areas with efficient local exhaust or extraction ventilation Adequate training and information should be provided to individual workers	Agriculture Electronic Medicines Medical technology Construction Automotive production Textiles Food processing Cosmetics	[44]
WHO	The Guideline Development Group (GDG) recommends assigning hazard classes to all MNMs according to the Globally Harmonized System (GHS) of Classification and Labelling of Chemicals for use in safety data sheets The GDG recommends updating safety data sheets with MNM-specific hazard information, or indicating which toxicological end-points did not have adequate testing available	Industries	[45]
FDA	Agglomeration and size distribution of nanomaterials under the conditions of toxicity testing and as expected in the final product In vitro and in vivo toxicological data on nanomaterial ingredients and their impurities, dermal penetration, potential inhalation, irritation (skin and eye) and sensitization studies, mutagenicity/genotoxicity studies.	Cosmetic industries	[46]

**Table 4:** Types of nanomaterials used in sensor fabrication for foodborne pathogens and their toxins detection.

Biosensors	Sensing Platform	Nanomaterials used in Biosensor Fabrication	Food Matrix	Pathogens/Toxins	Detection Limit	Analysis Time	Ref
Electrochemical biosensor	Screen printed carbon electrode	PLA-AuNPs (polylactic acid-stabilized gold nanoparticles)	Shellfish	Standard *Vibrio parahemolyticus*	2.16 × 10^−6^ μM	NS	[103]
Electrochemical DNA biosensor	Screen printed carbon electrode	PLA-AuNPs	Cockle	Standard *Vibrio parahemolyticus*	5.3 × 10^−12^	10 min	[104]
Paper-based biosensor	Gold electrode	Magnetic beads	Ground beef, Turkey sausage, Lettuce and Milk	Standard *Staphylococcus aureus*	40 cfu/mL	1 min	[105]
Aptamer-based biosensor	Gold electrode	Cys-PAMAM (cystamine-poly(amido-amine) dendrimers)	Peanuts	Aflatoxin B1	0.40 nM	10 min	[77]
Electrochemical immunosensor	Graphite electrode	Carboxylic acid-MWCNT (multiwalled carbon nanotubes)	Milk	Standard *Salmonella*, *Campylobacter* and *Escherichia coli*	400–800 cfu/mL	30 min	[106]
Electrochemical impedance Immunosensor	Glassy carbon electrode	AuNPs-MWCNT-PAMAM	Milk	Standard *Salmonella typhimurium*	5.0 × 10^2^ cfu/mL	NS	[107]
Lytic phage-based magnetoelastic biosensors	Iron-Nickel Base Magnetic ribbon	Cr-Au layer (Chromium)	Spinach Leaves	Standard *Staphylococcus aureus* (MRSA)	1.76 log cfu/25 mm^2^ surface of spinach	30 min	[108]
Amperometric immunosensing strips	Screen printed carbon electrode	AuNPs	Milk	Standard *Escherichia coli* O157:H7	50 cfu/strip in milk	1 h	[54]
Impedimetric biosensor	Gold disk electrodes	Pyrrole-3-carboxylic acid	Apple Juice	Standard *Salmonellatyphimurium*	3 cfu/mL	45 min	[102]
Amperometry biosensor	Glassy carbon disc electrode	Polypyrrole	NA	Laboratory isolates of *Listeria monocytogenes*	10^5^ cfu/mL	30 min	[109]
Colorimetric aptasensor	Magnetic beads	NA (not applicable)	Salmon	Standard *Vibrio parahemolyticus*	10^2^–10^7^ cfu/mL	NS	[110]
Fluorescence immunoassay	CdTe quantum dots (Cadmium telluride)	NA	Whole milk	Standard *Escherichia coli* O157:H7	5 × 10^2^–10^7^ cfu/mL	NS	[111]
Lateral flow biosensor	AuNPs (Gold nanoparticles)	NA	Milk	Standard *Salmonella enteriditis*	10^1^ cfu/mL	10 min	[112]
Mid-Infrared pathogen sensor	Magnetic nanoparticles	NA	Spinach and Milk	Standard bacterial cultures	10^4^–10^5^ cfu/mL	30 min	[113]
Aptamer-based biosensor	AuNPs	NA	Milk powder	Laboratory isolates of *Escherichia coli* O157:H7	10 cfu/mL	30 min	[114]
Electrochemical biosensor	Gold electrode	NA	Lettuce	Laboratory isolates of Norovirus	60 copies/mL	1 h	[55]
Gold nanoprobe	AuNPs	NA	NS (not specified)	Laboratory isolates of *Staphylococcus aureus*, *Listeria monocytogenes*, *Salmonella* spp.	123 fg/μL	30 min	[115]
Fluorometric graphene oxide-based assay	Graphene oxide	NA	NA	Standard *Salmonella enteriditis*	25 cfu/mL	NS	[116]
Chemiluminescent aptasensor	Fe_3_O_4_ GO NPs (Graphene oxide/iron nanoparticles)	NA	NA	Standard pre killed *Escherichia coli* O157:H7	4.5 × 10^3^ cfu/mL	1 h	[117]
Magnetoresistive-based immunoassay	Fe_2_O_3_ superparamagnetic particles	NA	NA	Standard Aflatoxins B1, Zearalenone	50 pg/mL	10 min	[118]
Goldnanoprobe-nucleic acid sequence-based amplification	Au colloid	NA	NA	Standard *Salmonella* strains	5 cfu/mL	80 min	[119]
Bioconjugate nanocapsules	AuNPs	NA	NA	*Listeria monocytogenes*	8.1 × 10^5^ cfu/ml and 2.6 × 10^7^ cfu/mL	5 min	[120]
Silicon-based DNA biosensor	Silicon wafer	NA	NS	Laboratory isolates of *Salmonella enteritidis*	1 ng/mL	NS	[85]
